# Effects of *Piper sarmentosum* Roxb. on hypertension and diabetes mellitus: A systematic review and meta-analysis

**DOI:** 10.3389/fphar.2022.976247

**Published:** 2022-08-25

**Authors:** Nur Syakirah Othman, Nur Aishah Che Roos, Amilia Aminuddin, Jaya Kumar Murthy, Adila A. Hamid, Azizah Ugusman

**Affiliations:** ^1^ Department of Physiology, Faculty of Medicine, Universiti Kebangsaan Malaysia, Kuala Lumpur, Malaysia; ^2^ Faculty of Medicine and Defence Health, National Defence University of Malaysia, Kuala Lumpur, Malaysia

**Keywords:** antihypertensive, hypertension, *Piper sarmentosum*, diabetes mellitus, antidiabetic

## Abstract

Hypertension and diabetes mellitus are among the most prevalent diseases affecting people from all walks of life. Medicinal herbs have garnered interest as potential agents for the prevention and treatment of diabetes mellitus and hypertension due to their multiple beneficial effects. *Piper sarmentosum* Roxb. (PS) is an edible medicinal plant that has been traditionally used in Asia for treating hypertension and diabetes mellitus. This review is aimed to provide comprehensive information from the literature on the effects of PS on hypertension and diabetes mellitus. A computerized database search was performed on Scopus, PubMed and Web of Science databases with the following set of keywords: Piper sarmentosum AND diabetes mellitus OR diabetic OR diabetes OR hyperglyc*emia OR blood glucose OR HbA1c OR glycated h*emoglobin OR h*emoglobin A1c OR hyperten* OR blood pressure. A total of 47 articles were screened and 14 articles published between the years 1998 until 2021 were included for data extraction, comprising of six articles on antihypertensive and eight articles on antidiabetic effects of PS. These studies consist of two *in vitro* studies and eleven *in vivo* animal studies. Meta-analysis of three studies on hypertension showed that PS versus no treatment significantly lowered the systolic blood pressure with mean difference (MD) −39.84 mmHg (95% confidence interval (CI) −45.05, −34.62; p < 0.01), diastolic blood pressure with MD −26.68 mmHg (95% CI −31.48, −21.88; p < 0.01), and mean arterial pressure with MD −30.56 mmHg (95% CI −34.49, −26.63; p < 0.01). Most of the studies revealed positive effects of PS against hypertension and diabetes mellitus, suggesting the potential of PS as a natural source of antidiabetic and antihypertensive agents.

## 1 Introduction

Hypertension and diabetes mellitus are among the most common non-communicable diseases and cardiovascular risk factors worldwide. Diabetic patients have a two to three-fold rise in hypertension prevalence compared to non-diabetics (Wan et al., 2018). Hypertension coexists in about 40–60% of type 2 diabetic patients (Tatsumi and Ohkubo, 2017). Hypertension may also precede the onset of diabetes, with more than 50% of adults having both hypertension and diabetes at the time of diagnosis (Nibouche and Biad, 2016). Diabetes mellitus and hypertension often coexist and share common pathways contributing to metabolic syndrome. Metabolic syndrome is associated with a greater risk for cardiovascular diseases (CVD) including heart attack and stroke (Isomaa et al., 2001; Tune et al., 2017).

The development of hypertension in diabetic patients is contributed by multiple factors such as insulin resistance, hyperglycemia, oxidative stress, and inflammation. Insulin resistance leads to the development of hyperinsulinemia. The anti-natriuretic activity of insulin increases sodium and water reabsorption from the renal tubules, leading to volume overload and elevation of blood pressure. Moreover, the body’s extracellular fluid volume rises because fluid move from the tissues into the vasculature following hyperglycemia-induced hyperosmolarity (Kawasoe et al., 2017). Hyperinsulinemia also activates the sympathetic nervous system and renin-angiotensin system (RAS), leading to an increase in blood pressure (Ohishi, 2018). Besides, hyperinsulinemia leads to vascular smooth muscle cell proliferation and increased vascular stiffness that predispose to hypertension (Shiny et al., 2016; Tsimihodimos et al., 2018). Furthermore, hyperglycemia triggers oxidative stress and inflammatory processes in the vascular wall that cause endothelial dysfunction, impaired vasodilatation and eventually hypertension (Wong et al., 2013; Oguntibeju, 2019; Sun et al., 2020).

On the other hand, hypertensive patients also have an increased risk of developing diabetes mellitus (Zhang et al., 2020). Hypertension is characterized by endothelial dysfunction, which might link hypertension with diabetes (Emdin et al., 2015). Numerous studies have reported that decreased endothelium-dependent vasodilatation in hypertension leads to decreased capillary recruitment that restricts insulin delivery to the metabolically active, insulin-sensitive muscle tissues (Bonadonna et al., 1998; Serné et al., 1999; Meigs et al., 2004). Besides, the altered endothelial permeability impairs insulin delivery to the interstitial space (Meigs et al., 2004). The interstitial insulin level is a rate-limiting step for insulin effectiveness (Miles et al., 1995).

Hypertension and diabetes mellitus share not only common pathophysiologic pathways but also common complications involving the macro- and microvascular disorders. Macrovascular complications include stroke, coronary artery disease and peripheral vascular disease, while microvascular complications include retinopathy, nephropathy and neuropathy (Yamazaki et al., 2018). Since the development of hypertension in patients with diabetes is marked by a significant risk of macro- and microvascular complications, efforts should be made to delay or ideally prevent the increase in blood pressure. Hence, a therapy that can help with glycemic and blood pressure control will be of significant clinical value.

**FIGURE 1:**
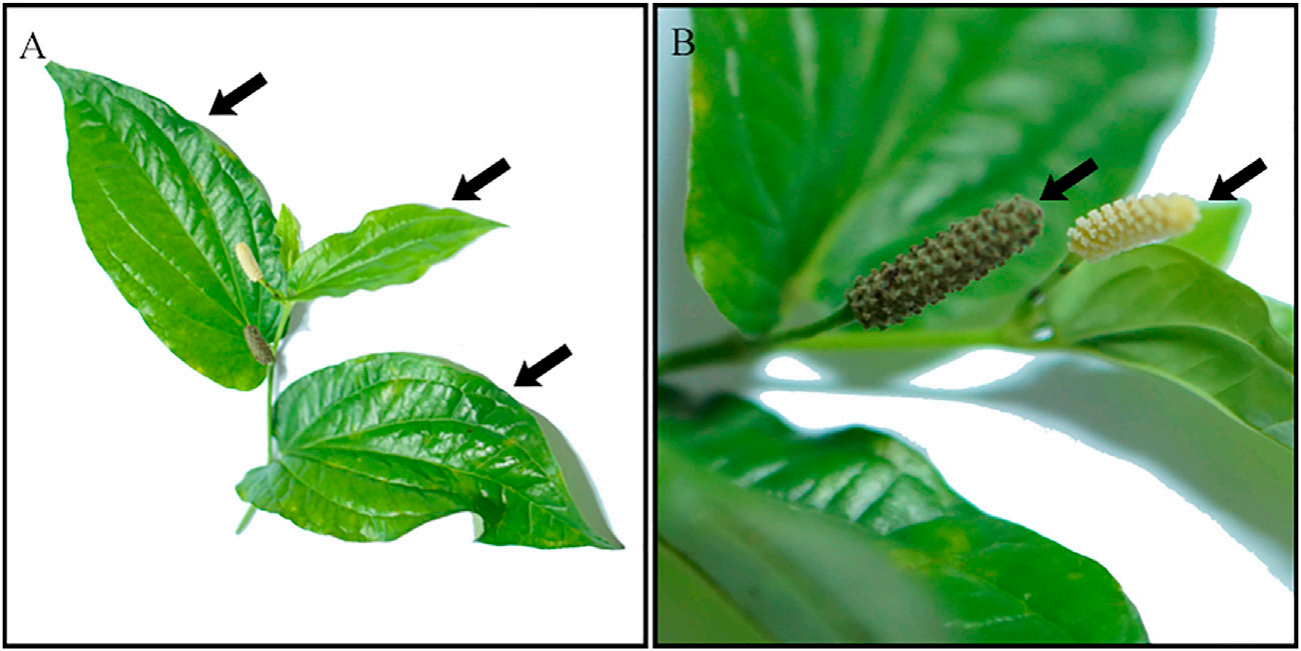
*Piper sarmentosum* Roxb. Leaves **(A)** and fruit **(B)**.

*Piper sarmentosum* Roxb. (PS) is a herbaceous plant that is widely cultivated in Southeast Asia, Northeast India and China (Mathew et al., 2004). It is a terrestrial creeping herb that belongs to the family of Piperaceae, with an average height of 20 cm and easily grows in shady areas (Mohd Zainudin et al., 2013). PS leaves are light to dark green in colour (Chaveerach et al., 2006), and the fruits are obovoid in shape and sweet to taste (Figure 1). PS are also known as “Kaduk” or “Sirih duduk” in Malaysia; “Cha plu” in Thailand; “Karuk”, “Mengkadak” or “Sirih tanah” in Indonesia; “La lot” in Vietnam; “Phak i leut” in Laos and “Jia ju”, “Xi ye qing wei teng” or “Qing ju” in China (Ismail et al., 2018).

Historically, PS has long been used as a culinary plant as well as a traditional medicine to treat hypertension, joint aches (Subramaniam et al., 2003), cough, pleurisy, fever (Farhana Syed Ab Rahman 2016), and indigestion (Hussain et al., 2012). Pharmacologically, PS possesses antiatherosclerosis (Amran et al., 2010; Amran et al., 2011), anticarcinogenic (Zainal Ariffin et al., 2009), anti-inflammatory (Ridtitid et al., 2007; Zakaria et al., 2010), antiplatelet aggregation (Han, 1995), antiangiogenic (Hussain et al., 2015) and antituberculosis (Hussain et al., 2008) effects. PS also protects against glucocorticoid-induced osteoporosis (Mohamad Asri et al., 2016) and paracetamol-induced oxidative liver injury (Nur Azlina et al., 2014). Several studies have reported that PS has a high antioxidative activity (Subramaniam et al., 2003; Hafizah et al., 2010; Ugusman et al., 2010; Mohd Zainudin et al., 2015). Interestingly, PS also has antidiabetic (Peungvicha et al., 1998; Azlina et al., 2009; Luangpirom et al., 2014) and antihypertensive effects (Hussan et al., 2013; Alwi et al., 2018; Fauzy et al., 2019).

Previous studies demonstrated that different parts of PS contain various phytochemical compounds. The methanolic extract of PS leaves contains carotenes, tannin, vitamin C, vitamin E, xanthophylls, and phenolics (Chanwitheesuk et al., 2005), while the roots and stems of PS contain piplartine, langkamide and 3,4,5-trimethoxycinnamic acid (Bokesch et al., 2011). Besides, the aqueous extract of PS leaves contains flavonoids, phenolic and ascorbic acids (Sumazian et al., 2010). Meanwhile, three amides; 3-(3′,4′,5′-trimethoxyphenylpropanoyl) pyrrolidine, 3-(4′-methoxyphenylpropanoyl) pyrrole and N-(3-phenylpropanoyl) pyrrole, and a sterol; β-sitosterol have been successfully isolated from the hexane and ethyl acetate extracts of aerial parts of PS (Atiax et al., 2011).

The aqueous extract of PS leaves up to 2000 mg/kg/day does not cause subacute toxicity effects; hence it is safe for consumption (Mohd Zainudin et al., 2013). Recently, the consumption of herbal products for complementary and alternative medicine has been rapidly increasing. Previous studies have suggested that antioxidants could be beneficial for managing diabetes mellitus and hypertension (Sabadashka et al., 2021; Tain and Hsu 2022). An earlier systematic review has confirmed the antioxidative effect of PS (Ismail et al., 2018). However, no systematic review and meta-analysis have been conducted to explore the effect of PS on hypertension and diabetes. Thus, we aimed to systemically review the available literature on the effects of PS on diabetes mellitus and hypertension to better understand the herb’s medicinal potential to support its scientific use further.

## 2 Methodology

### 2.1 Search strategy

The review was reported according to the Preferred Reporting Items for Systematic Reviews and Meta-Analyses (PRISMA) guideline. The relevant studies were identified from three main databases: Scopus, PubMed and Web of Science (WoS) from their respective inception dates to March 2022. The last search was performed on 26th March 2022. The following set of keywords was used: Piper sarmentosum AND (diabetes mellitus OR diabetic OR diabetes OR hyperglyc*emia OR blood glucose OR HbA1c OR glycated h*emoglobin OR h*emoglobin A1c OR hyperten* OR blood pressure). Articles that could be missing during the database search were searched from the reference list of the review articles retrieved from the initial search and added to the selected articles list (Thent and Das, 2015). The protocol of this review was registered at the International Platform of Registered Systematic Review and Meta-analysis Protocols (INPLASY registration number: 202240020) (Othman et al., 2022).

### 2.2 Eligibility and exclusion criteria

Only full-length original research articles published in the English language were included. Any clinical (randomized controlled trial) and preclinical (*in vitro*, *in vivo*, and *ex vivo*) studies that reported the effects of PS on hypertension and diabetes models, regardless of the route of administration, formulation, dose and duration of intervention were included. However, any observational studies and studies using combined preparation of PS with other herbs were excluded. Review articles, news, book chapters, conference proceedings, editorial letters, and case studies were also excluded from this review.

### 2.3 Study selection and data extraction

The literature search and articles screening were performed according to the population, interventions, compare, outcome and study design (PICOS) framework, as follows:

1) Population (P): Adult patients with established hypertension and/or diabetes and preclinical models of hypertension and diabetes, regardless of animal species, were included.
2) Intervention (I): Studies that used PS as an intervention in the experimental group were included.
3) Comparison (C): The comparator groups received either no intervention or were treated with relevant conventional drug.
4) Outcome (O): Changes in blood pressure, blood glucose or glycosylated hemoglobin (HbA1_C_).
5) Study design (S): Clinical (randomized controlled trial) and preclinical (*in vitro*, *in vivo*, *ex vivo*) studies.

The articles were primarily screened through the articles’ type, language, title and abstracts related to the effect of PS on hypertension and diabetes. Duplicates were removed from each database. The secondary screening involved the removal of articles based on the inclusion and exclusion criteria set for this study. Any similar studies were removed to avoid selection bias. The retrieved articles were reviewed independently by two authors (NO. and AU). Any disagreements were resolved by seeking a third reviewer’s opinion (NR).

Study characteristics including study design, animal model used, plant source, the part of plant used, type of extraction and phytochemical used were extracted. Primary outcomes such as systolic blood pressure (SBP), diastolic blood pressure (DBP), mean arterial pressure (MAP), and blood glucose level were extracted. Other parameters such as nitric oxide (NO), endothelial nitric oxide synthase (eNOS), asymmetric dimethylarginine (ADMA), endothelin-1 (ET-1) and malondialdehyde (MDA) levels, α-glucosidase and α-amylase activities, insulin and urine glucose levels, body and organ weights, and histological analysis of the target organs were also extracted where available. For studies with more than one interventional arms, data from only the relevant arms were considered, e.g. hypertensive rats receiving PS versus hypertensive rats receiving positive control or no treatment. In case of missing or incomplete information, the respective author was contacted by email and the missing data were requested if necessary.

### 2.4 Risk of bias assessment

Two reviewers analyzed the risk of bias independently (NO and AU). Any disagreement was resolved through discussion with the third reviewer (NR). Cochrane risk of bias (RoB) tool was used to assess the risk of bias in randomized clinical trials (Higgins et al., 2019). Meanwhile, animal studies were assessed using the Systematic Review Center for Laboratory Animal Experimentation (SYRCLE) risk of bias tool. The main components of this item were as follows: 1) Selection bias: random sequence generation, baseline characteristics, allocation concealment; 2) Detection bias: random housing, blinding, random outcome assessment; 3) Attrition bias: incomplete outcome data; 4) Reporting bias: selective reporting; and 5) Other bias (Hooijmans et al., 2014). For *in vitro* studies, a customized risk of bias tool based on the Joanna Briggs Institute (JBI) checklist for non-randomized experimental studies were used (JBI, 2020). The customized RoB tool comprises of three domains as follows: 1) Reporting quality: source of plant, amount of plant/extract/sample used; 2) Performance bias: reliable tools and/or reagents used to measure outcome; and 3) Detection bias: standard/appropriate control used, multiple measurements of outcome performed. Each domain was evaluated as being a high, moderate, low or unclear risk of bias.

### 2.5 Statistical analysis

The meta-analysis was performed using the Review Manager (RevMan) 5.4 software (The Cochrane Collaboration, 2020). The mean difference (MD) together with its 95% confidence intervals (CI) was used in reporting the effect size of PS on blood pressure (BP). The heterogeneity between studies was evaluated using 1) the Chi-squared test with a p-value of less than 0.10 denoted statistical significance and 2) the Higgin’s I^2^ statistic (Higgins et al., 2003). An I^2^ value of less than 25% was regarded as low heterogeneity, 30%–50% was regarded as moderate heterogeneity, and any value above 75% as high heterogeneity. Due to the small number of studies available for meta-analysis, a fixed-effect (FE) model was used. A p-value of less than 0.05 indicated statistical significance. Sensitivity analysis was conducted by only including studies using a similar PS dose (500 mg/kg) for evaluation of result’s robustness. No subgroup analysis was performed due to the limited number of studies available for meta-analysis. A funnel plot was not reported as less than ten studies were included in the meta-analysis.

## 3 Results

### 3.1 Studies selected

**FIGURE 2:**
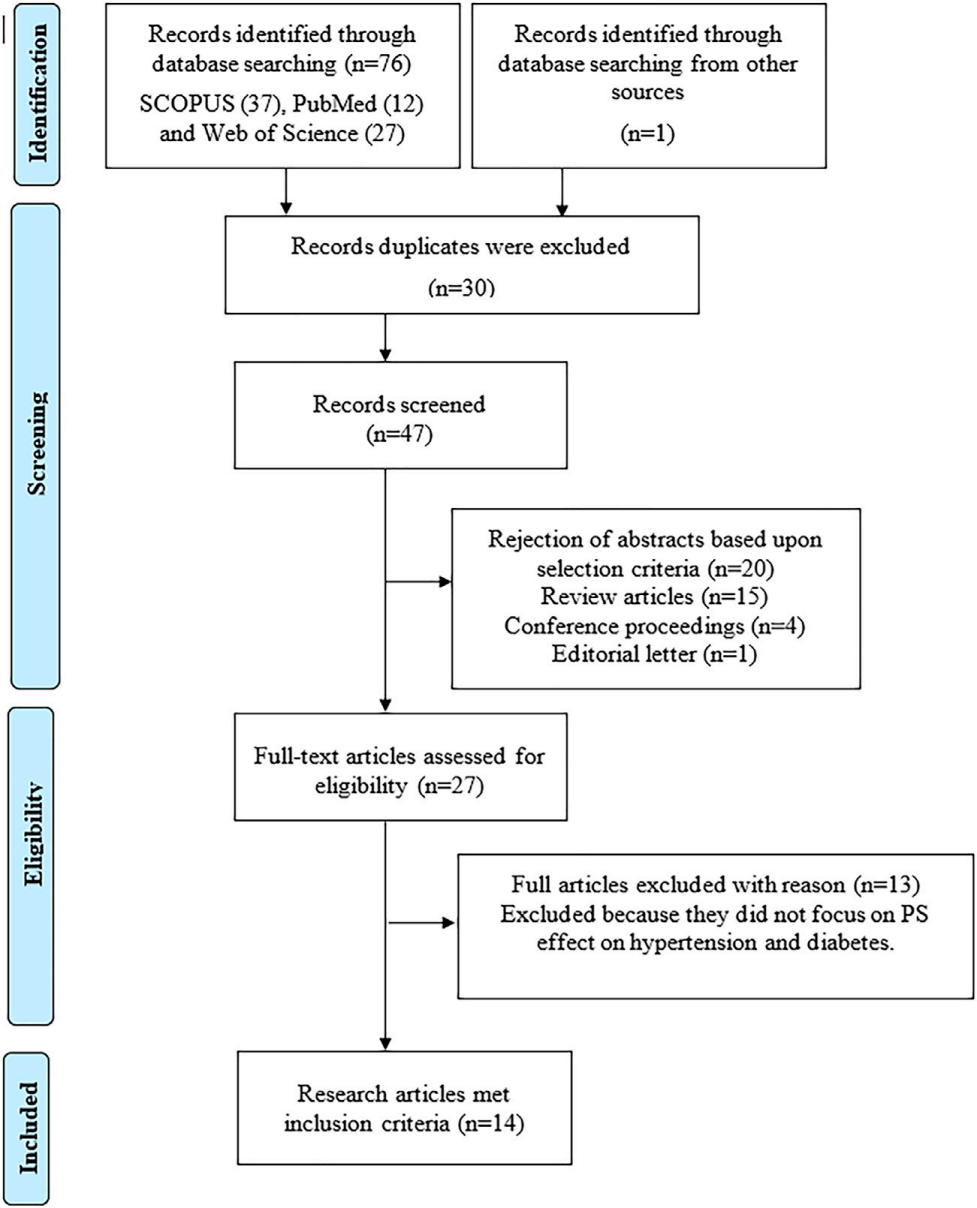
The selection process of the articles according to the Preferred Reporting Items for Systematic Reviews and Meta-Analyses (PRISMA) guideline.

Initially, a total of 76 potential articles were identified in Scopus (*n* = 37), PubMed (*n* = 12) and Web of Science (*n* = 27). An additional article was retrieved from the list of references cited in the review articles (*n* = 1). Subsequently, 30 articles were removed due to duplication. After reviewing the titles and abstracts, 33 articles were excluded. The full text of the remaining 27 articles were read thoroughly and 13 articles were further excluded as they did not fulfil the inclusion criteria. Finally, 14 studies published between the years 1998–2021 were selected for inclusion in this review, comprising of six articles on antihypertensive and eight articles on antidiabetic effects of PS. A flowchart of the article selection process is shown in Figure 2.

### 3.2 Risk of bias

**FIGURE 3:**
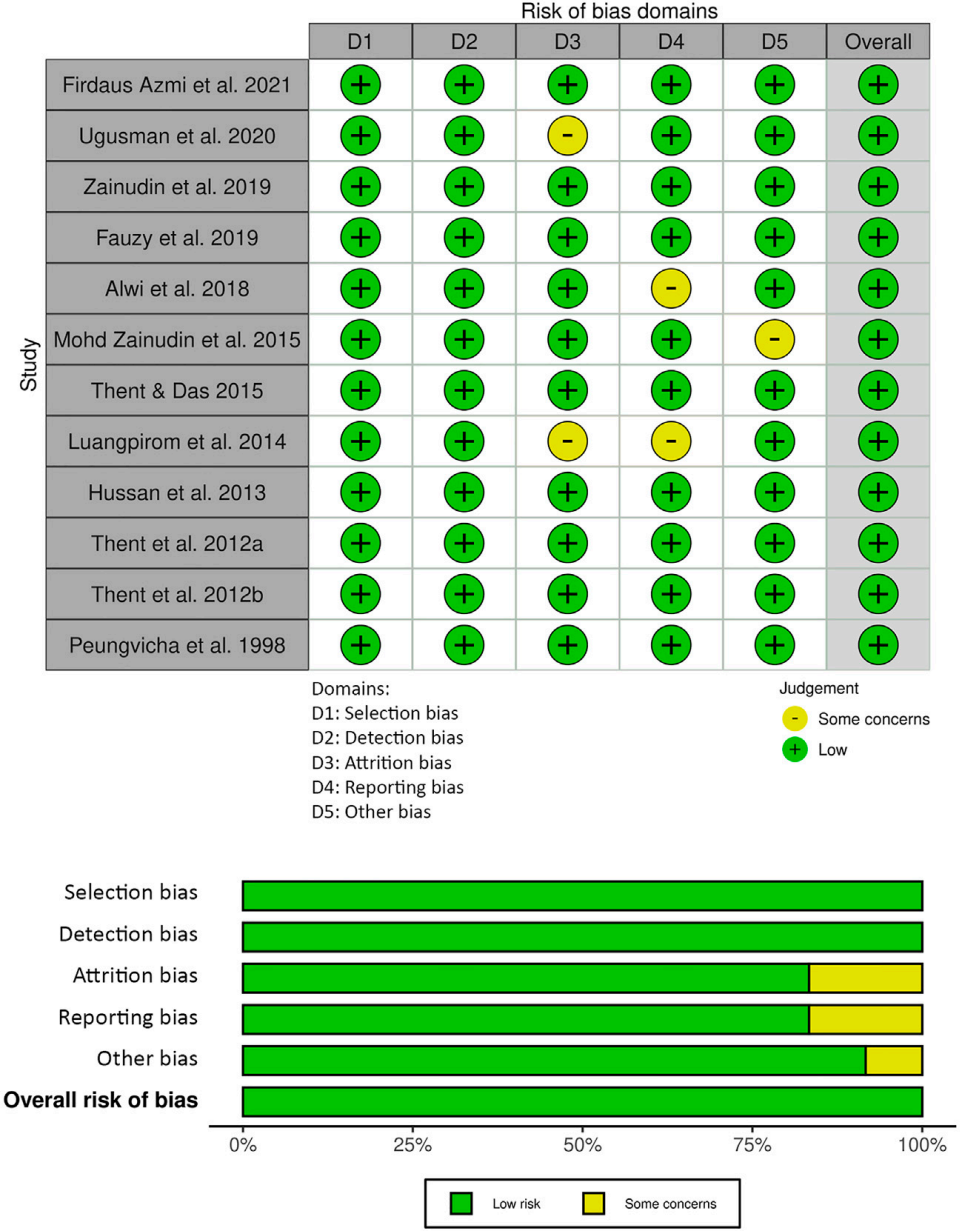
Systematic Review Center for Laboratory Animal Experimentation (SYRCLE) risk of bias summary: review authors’ judgements about each risk of bias item for each included study.

**FIGURE 4:**
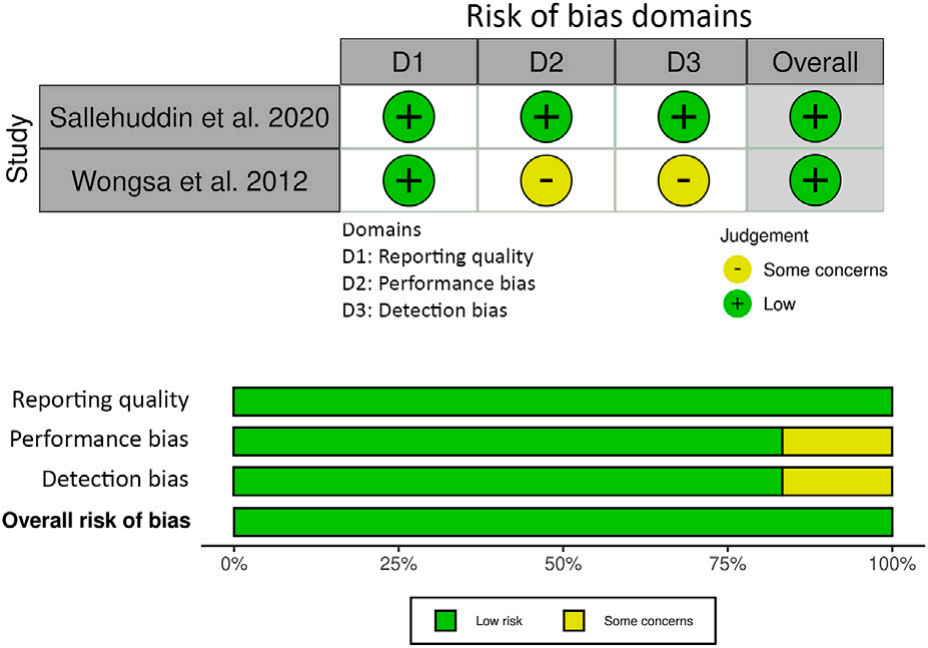
The Joanna Briggs Institute (JBI) critical appraisal checklist summary for reporting *in vitro* studies.

The risk of bias assessment for the animal studies included in this review is presented in Figure 3. Selection risk and detection of bias was low for all studies. Attrition bias was either low risk (*n* = 10) or unclear (*n* = 2) in the included studies. Selection reporting risk of bias was unclear in two of the studies and the remainder (*n* = 10) was low risk. Other bias was unclear in one of the studies and low for the remainder (*n* = 11). The JBI critical appraisal checklist summary for reporting *in vitro* studies is shown in Figure 4. Reporting quality bias was low for all studies. Performance and detection bias was either low risk (*n* = 1) or unclear (*n* = 1).

### 3.3 Study design characteristics

The characteristics of the selected studies are described in Tables 1, 2. All the included studies were preclinical studies, with no clinical trial done previously. As for the preclinical studies, 12 studies involved animal models (Peungvicha et al., 1998; Thent et al., 2012a; Thent et al., 2012b; Hussan et al., 2013; Luangpirom et al., 2014; Mohd Zainudin et al., 2015; Thent and Das, 2015; Alwi et al., 2018; Mohd Zainudin et al., 2019; Fauzy et al., 2019; Ugusman et al., 2020; Firdaus Azmi et al., 2021) and the remaining two studies were *in vitro* chemical assay studies (Wongsa et al., 2012; Sallehuddin et al., 2020). For studies related to the effect of PS on hypertension, the animal models of hypertension used were spontaneous hypertensive rats (SHR) (Mohd Zainudin et al., 2015; Fauzy et al., 2019; Mohd Zainudin et al., 2019), dexamethasone-induced hypertensive rats (Ugusman et al., 2020; Firdaus Azmi et al., 2021) and N^ω^-nitro-l-arginine methyl ester hydrochloride (l-NAME)-induced hypertensive rats (Alwi et al., 2018). Meanwhile, induction of diabetes in all the studies were done through streptozotocin (STZ) injection.

**TABLE 1:** Characteristics of selected studies on the effects of *Piper sarmentosum* Roxb. on hypertension.

Study design	Plant source	Plant part	Type of extract	Phyto-chemical(s)	Results	Outcomes	References
*In vivo* animal study. Thirty male Sprague Dawley rats (8 weeks old) were divided into five groups (*n* = 6) including control (normal saline), PS (500 mg/kg/day orally), dexamethasone (20 μg/kg/day subcutaneously to induce hypertension), dexamethasone + PS and dexamethasone + captopril (40 mg/kg/day orally as positive control). Treatments were administered for 28 days. The systolic blood pressure (SBP), diastolic blood pressure (DBP) and mean arterial pressure (MAP) of the rats were measured using tail-cuff method.	Selangor and Penang, Malaysia	Leaf	Aqueous	Rutin Vitexin	PS decreased SBP at day 14 (132.72 ± 3.07 mmHg vs. 109.28 ± 2.95 mmHg, p < 0.001) and day 28 (143.06 ± 3.65 mmHg vs. 105.22 ± 2.89 mmHg, p < 0.001), DBP at day 14 (105.28 ± 2.58 mmHg vs. 86.56 ± 5.31 mmHg, p < 0.05) and day 28 (104.61 ± 2.32 mmHg vs. 82.83 ± 3.49 mmHg, p < 0.001), and MAP at day 14 (114.43 ± 2.3 mmHg vs. 94.13 ± 4.41 mmHg, p < 0.05) and day 28 (117.43 ± 2.06 mmHg vs. 90.30 ± 2.53 mmHg, p < 0.001) and these effects were comparable to captopril.	PS extract quantified to rutin and vitexin has antihypertensive effect.	Firdaus Azmi et al. (2021)
*In vivo* animal study. Thirty male Sprague Dawley rats (8–12 weeks) were divided into five groups (*n* = 6) including control (normal saline), PS (500 mg/kg/day orally), dexamethasone (20 μg/kg/day subcutaneously to induce hypertension), dexamethasone + PS and dexamethasone + captopril (40 mg/kg/day orally as positive control). Treatments were administered for 28 days. The SBP, DBP and MAP of the rats were measured using tail-cuff method. The rat’s thoracic aorta was analyzed for endothelial nitric oxide synthase (eNOS) mRNA expression, protein and activity while the serum was analyzed for nitric oxide (NO) level.	Penang, Malaysia	Leaf	Aqueous	Rutin Vitexin	- PS decreased SBP at day 14 (136 ± 2.0 mmHg vs. 111 ± 2.8 mmHg, p < 0.001) and day 28 (146 ± 2.7 mmHg vs. 107 ± 2.9 mmHg, p < 0.001), DBP at day 14 (105 ± 2.5 mmHg vs. 82 ± 5.1 mmHg, p < 0.01) and day 28 (104 ± 3.1 mmHg vs. 79 ± 1.7 mmHg, p < 0.01), and MAP at day 14 (114.43 ± 2.2 mmHg vs. 92 ± 3.7 mmHg, p < 0.001) and day 28 (118 ± 2.5 mmHg vs. 88 ± 1.4 mmHg, p < 0.001) and these effects were comparable to captopril. - Treatment of dexamethasone-induced hypertensive rats with PS increased the NO level (31.0 ± 5.18 µM vs. 56.8 ± 6.22 µM, p < 0.05), eNOS mRNA expression by 1.4 folds (p < 0.01), eNOS protein (10.17 ± 1.54 pg/mg protein vs. 40.17 ± 9.51 pg/mg protein, p < 0.01), and eNOS activity (1.1 ± 0.073 µM nitrite/min/mg protein vs. 1.4 ± 0.109 µM nitrite/min/mg protein, p < 0.05).	PS demonstrates antihypertensive effect by enhancing eNOS activity and production of NO.	Ugusman et al. (2020)
*In vivo* animal study. Twenty-four spontaneous hypertensive rats (SHR) were divided into four groups (*n* = 6) including hypertensive control (distilled water), PS (500 mg/kg/day), perindopril (3 mg/kg/day), and combined PS (500 mg/kg/day) + perindopril (1.5 mg/kg/day). All treatments were given orally for 28 days. SBP and DBP were measured using tail-cuff method. Serum NO and plasma asymmetric dimethylarginine (ADMA) levels were also determined.	Kuantan, Pahang, Malaysia	Leaf	Aqueous		- PS decreased SBP (p < 0.05) and DBP (p < 0.05) of SHR. The reduction in DBP was greater than SBP (p < 0.05). However, PS was not as potent as perindopril in reducing blood pressure. - There was a reduction in plasma ADMA level (p < 0.05) and an increase in serum NO level (p < 0.05) in SHR following PS treatment.	PS reduces blood pressure by increasing the clearance of ADMA and production of NO.	Mohd Zainudin et al. (2019)
*In vivo* animal study. Twenty-four SHR (11 weeks) were divided into four groups (*n* = 6): hypertension control (distilled water), PS (500 mg/kg/day), perindopril (3 mg/kg/day), and combined PS (500 mg/kg/day) + perindopril (1.5 mg/kg/day). All treatments were given orally for 28 days. SBP, DBP and MAP were measured using tail-cuff method. Rats mesenteric arteries were analyzed for NO and endothelin-1 (ET-1) levels.	Kuantan, Pahang, Malaysia	Leaf	Aqueous		- SHR showed reductions in SBP (p < 0.05), DBP (p < 0.05) and MAP (p < 0.05) with PS treatment. However, PS showed no superior effect in reducing blood pressure compared to perindopril. - PS increased mesenteric artery NO level (p < 0.05) and reduced ET-1 level (p < 0.05) in SHR.	PS decreases blood pressure by reducing ET-1 level and increasing NO level in the resistance artery.	Fauzy et al. (2019)
*In vivo* animal study. Thirty-six Wistar rats (6–8 weeks) were divided into six groups (*n* = 6): control (normal saline), PS (500 mg/kg/day), l-NAME (100 mg/L) to induce hypertension) and three groups of combined l-NAME and different doses of PS (125, 250 and 500 mg/kg/day). Treatments were administered orally for 4 weeks. SBP, DBP and MAP were measured using tail-cuff method. Serum NO and malondialdehyde (MDA) levels were quantitated.	Selayang, Selangor, Malaysia	Leaf	Aqueous		- Treatment with three doses of PS (125, 250 and 500 mg/kg/day) lowered the SBP (172.3 ± 5.06 mmHg vs. 126.0 ± 5.2, 127.83 ± 3.79 and 129.67 ± 3.74 mmHg, p < 0.001), DBP (127.5 ± 3.93 mmHg vs. 84.5 ± 4.38, 90.0 ± 2.44, and 86.3 ± 4.19 mmHg, p < 0.05), and MAP (142.0 ± 4.49 mmHg vs. 97.0 ± 3.44, 101.2 ± 1.86 and 100.5 ± 2.71 mmHg, p < 0.05). - There were decreased serum MDA (65.59 ± 5.46 nmol/g protein vs. 22.70 ± 3.63, 16.57 ± 4.64, and 25.15 ± 11.39 nmol/g protein, p < 0.001) and increased serum NO level (4.5 ± 1.92 μM vs. 56.33 ± 9.15, 80.88 ± 8.55 μM, and 75.02 ± 8.46 μM, p < 0.001).	Antihypertensive effect of PS is mediated by increased NO and reduced oxidative stress.	Alwi et al. (2018)
*In vivo* animal study. Six normotensive Wistar rats served as negative control group, while thirty-two SHR (10 weeks) were divided into four groups (n = 6 for positive control, n = 8 for three treatment groups): hypertensive control, and SHR treated with three different doses of PS (0.5, 1, 2 mg/kg/day) orally for 28 days. The blood pressure, serum NO, MDA and total cholesterol levels were measured.	Kuantan, Pahang, Malaysia	Leaf	Aqueous		- PS reduced SBP (p < 0.05), DBP (p < 0.05) and MAP (p < 0.05). - PS increased serum NO (p < 0.05) and decreased MDA (p < 0.05) levels.	PS attenuates hypertension by increasing NO level and decreasing oxidative stress.	Mohd Zainudin et al. (2015)

ADMA, plasma asymmetric dimethylarginine; eNOS, endothelial nitric oxide synthase; ET, Endothelin-1; DBP, diastolic blood pressure; MAP, mean arterial pressure; l-NAME, N^ω^-nitro-l-arginine methyl ester hydrochloride; MDA , malondialdehyde; NO, nitric oxide; PS, *Piper sarmentosum* Roxb.; SBP, systolic blood pressure; SHR, spontaneous hypertensive rats.

**TABLE 2:** Characteristics of selected studies on the effects of *Piper sarmentosum* Roxb. on diabetes mellitus.

Study design	Plant source	Plant parts	Type of extract	Phyto-chemical(s)	Results	Outcomes	References
*In vitro* study. Antidiabetic activity was measured using α-glucosidase inhibitory activity.	Selangor, Malaysia	Leaf	Ethanol	Catechin. Naringin	PS at 1,000 μg/ml did not inhibit α-glucosidase activity.	PS has no antidiabetic activity.	Sallehuddin et al. (2020)
*In vitro* study. Antidiabetic activity was evaluated by α-glucosidase and α-amylase inhibitory activity.	Chiangrai province, Thailand	Leaf	Aqueous	Caffeic acid p-Coumaric acid.	PS showed α-glucosidase inhibitory activity but did not show inhibition against α -amylase activity.	PS has antidiabetic effect by inhibiting α-glucosidase activity.	Wongsa et al. (2012)
*In vivo* animal study. Twenty-four male, Sprague Dawley rats were divided into four groups (n = 6) including non-diabetic control, non-diabetic treated with PS (0.125 g/kg/day), untreated diabetic (induced with single intramuscular injection of 50 mg/kg STZ) and diabetic treated with PS (0.125 g/kg/day). Treatment with PS was started 4 weeks after STZ injection, for a total of 28 days via intragastric tube. SBP was measured using tail-cuff method. The rat’s liver was collected for morphological analysis.	Negeri Sembilan, Malaysia	Leaf	Aqueous		-PS decreased the SBP of streptozotocin-induced diabetic rats (p < 0.05). -Compared to untreated diabetic rats, treatment with PS increased the liver weight (6.03 ± 0.39 g vs. 10.23 ± 0.27 g, p < 0.05) and reversed the diabetes-induced degenerative changes in the liver tissues as evidenced by absence of nuclear deformation in the of hepatocytes, less hyperemic areas in the sinusoids and less necrosis and vacuolization in the liver.	PS has a positive effect on diabetes and its complications.	Thent and Das (2015)
*In vivo* animal study. Fifty adult male mice (ICR strain, 8-week-old) were randomly divided into five groups (*n* = 6 for References group and *n* = 11 for diabetic groups) including non-diabetic control, untreated diabetic (induced with intraperitoneal injection of 6 mg/100 g BW STZ), diabetic treated with glibenclamide (1 mg/100 g BW/day orally as positive control), diabetic treated with PS_1_ (60 mg/100 g BW/day orally) and diabetic treated with PS_2_ (100 mg/100 g BW). Treatments were administered for 21 days. Fasting blood glucose (FBG) level was measured by glucometer via blood from the tail artery. Plasma was analysed for insulin levels. Pancreas was also assessed histologically.	Khon Kaen Province, Thailand	Leaf	Aqueous		Compared with untreated diabetic rats, diabetic rats treated with PS had: - Greater change in FBG (+46.01% vs. -32.75%). - Higher insulin level (14.19 ± 2.95 IU/L vs. 21.36 ± 2.53 IU/L, p < 0.05). - Increase in size and decrease in number of dead cells in the pancreatic islets. - PS was as potent as glibenclamide in increasing insulin level.	PS has hypoglycemic activities by increasing insulin secretion and improving pancreatic islet function.	Luangpirom et al. (2014)
*In vivo* animal study. Eighteen male Sprague Dawley rats were randomly divided into three groups (*n* = 6) including nondiabetic control, untreated diabetic (induced with single intramuscular injection of 50 mg/kg STZ) and diabetic treated with PS (0.125 g/kg/day orally). Treatment with PS was started 10 days following STZ induction and continued for 28 days. Body weight and kidney weight index were recorded. FBG was measured using glucometer from the tail vein. Kidneys were collected for histomorphometric and histological analysis.	Selangor, Malaysia	Leaf	Aqueous		- PS did not have significant effect on the body weight, kidney weight index and FBG of diabetic rats. - PS attenuated the histological changes in the diabetic rat’s kidney as evidenced by less contracted glomeruli, mild inflammatory cells infiltration, reduced urinary space size and absence of glomerular membrane thickening.	Antihyperglycemic activity of PS prevents further progression of diabetic nephropathy.	Hussan et al. (2013)
*In vivo* animal study. Twenty-four male Sprague Dawley rats were randomly divided into four groups (*n* = 6) including nondiabetic control (normal saline), nondiabetic treated with PS (0.125 g/kg/day orally), untreated diabetic (induced with single intramuscular injection of 50 mg/kg STZ) and diabetic treated with PS (0.125 g/kg/day orally). Treatments were administered for 28 days. The rat’s cardiac and aortic tissues were collected for histological analysis.	Negeri Sembilan, Malaysia	Leaf	Aqueous		Treatment of diabetic mice with PS caused less degenerative changes in the myocardium and aortic tissues as evidenced by lack of connective tissue deposit in the myocardium, reduced tunica media thickness, reduced tunica intima to tunica media ratio and less disruption of elastic fibre in the tunica media layer of the aorta.	PS has beneficial effect on diabetes by reducing degenerative changes in the myocardium and aorta.	Thent et al. (2012a)
*In vivo* animal study Thirty-two male Sprague Dawley rats were randomly divided into four groups (n = 8) including nondiabetic control (normal saline), nondiabetic treated with PS (0.125 g/kg/day orally), untreated diabetic (induced with single intramuscular injection of 50 mg/kg STZ) and diabetic treated with PS (0.125 g/kg/day orally). Treatments were started 28 days following diabetes induction for 28 days continuously. The body weight was recorded. Urine and blood glucose levels were measured by Combur test and glucometer, respectively. The rat’s left ventricular cardiac tissue and proximal aorta were analyzed under the electron microscope.	Negeri Sembilan, Malaysia	Leaf	Aqueous		Compared to untreated diabetic rats, PS supplementation to diabetic rats caused: - Higher body weight (178 ± 10.91 g vs. 231 ± 13.52 g, p < 0.05). - Lower FBG level (31.9 ± 1.72 mmol/L vs. 23.2 ± 2.24 mmol/L, p < 0.05). - Lower urine glucose level. - Less irregular arrays of myofibrils within the cardiac sarcomere. - Less disrupted cardiac muscle fibres. - Intact cardiac mitochondria with reduced mitochondrial size and cytoplasmic spaces. - Less disruption of the elastic lamina, decreased proliferation of smooth muscle cells and presence of the endothelial cells in the proximal aorta.	PS has antidiabetic effect and restores ultrastructural integrity of the diabetic cardiovascular tissues.	Thent et al. (2012b)
*In vivo* animal study. Male Wistar rats (5 weeks old) were divided into five groups (*n* = 6–8) including nondiabetic control, untreated diabetic (induced with single intraperitoneal injection of 75 mg/kg STZ), diabetic treated with glibenclamide (5 mg/kg orally), diabetic treated with PS_1_ (0.125 g/kg orally) and diabetic treated with PS_2_ (0.250 g/kg orally). Treatments were administered for 7 days. Fasting plasma glucose level and oral glucose tolerance test (OGTT) were determined.	Bangkok, Thailand	Whole plant	Aqueous, methanol soluble fraction and methanol insoluble fraction of aqueous extract.		- Single dose of PS did not reduce the blood glucose but repeated administration of 0.125 g/kg PS for 7 days produced significant decrease in the plasma glucose of diabetic rats. - Hypoglycemic effect of the methanol soluble fraction of PS aqueous extract was more potent than the aqueous extract.	PS has hypoglycemic effect.	Peungvicha et al. (1998)

FBG, fasting blood glucose; OGTT, oral glucose tolerance test; PS, *Piper sarmentosum* Roxb.; SBP, systolic blood pressure; STZ, streptozotocin.

The quality control and chemical analysis of PS extracts in the selected studies were summarized in Table 3. The origins of PS were from two main countries: Malaysia (Thent et al., 2012a; Thent et al., 2012b; Hussan et al., 2013; Mohd Zainudin et al., 2015; Thent and Das, 2015; Alwi et al., 2018; Fauzy et al., 2019; Mohd Zainudin et al., 2019; Sallehuddin et al., 2020; Ugusman et al., 2020; Firdaus Azmi et al., 2021) and Thailand (Peungvicha et al., 1998; Wongsa et al., 2012; Luangpirom et al., 2014). Most of the studies used the leaves of PS (Thent et al., 2012a, 2012b; Wongsa et al., 2012; Hussan et al., 2013; Luangpirom et al., 2014; Mohd Zainudin et al., 2015; Alwi et al., 2018; Fauzy et al., 2019; Mohd Zainudin et al., 2019; Sallehuddin et al., 2020; Ugusman et al., 2020; Firdaus Azmi et al., 2021) and only one study used all parts of PS (Peungvicha et al., 1998). Different types of PS extract were used, including ethanol extract (Thent and Das 2015; Sallehuddin et al., 2020), aqueous extract (Peungvicha et al., 1998; Thent et al., 2012a; Thent et al., 2012b; Wongsa et al., 2012; Hussan et al., 2013; Luangpirom et al., 2014; Mohd Zainudin et al., 2015; Alwi et al., 2018; Fauzy et al., 2019; Mohd Zainudin et al., 2019; Ugusman et al., 2020; Firdaus Azmi et al., 2021) and methanol-soluble and -insoluble fractions of aqueous extract of PS (Peungvicha et al., 1998). Among the active compounds found in the phytochemical analyses of the PS extracts were rutin, vitexin (Ugusman et al., 2020; Firdaus Azmi et al., 2021), catechin, naringin (Sallehuddin et al., 2020), caffeic acid and p-coumaric acid (Wongsa et al., 2012).

**TABLE 3:** Quality control and chemical analysis of *Piper sarmentosum* Roxb. extracts in the selected studies.

Study	Source	Concentration (%)	Quality control reported? (Yes/No)	Chemical analysis reported? (Yes/No)
Firdaus Azmi et al. (2021)	Selangor and Penang, Malaysia	10	Yes- Standardization based on active compounds	Yes-UPLC
Ugusman et al. (2020)	Penang, Malaysia	10	Yes-Standardization based on active compounds	Yes-HPLC
Mohd Zainudin et al. (2019)	Kuantan, Pahang, Malaysia	10	Yes-FRAP and DPPH radical scavenging assays	Yes-HPLC
Fauzy et al. (2019)	Kuantan, Pahang, Malaysia	10	No	No
Alwi et al. (2018)	Selayang, Selangor, Malaysia	10	Yes-Protocol citation	No
Mohd Zainudin et al. (2015)	Kuantan, Pahang, Malaysia	10	Yes- DPPH radical and superoxide scavenging assays	No
Sallehuddin et al. (2020)	Selangor, Malaysia	10	Yes- DPPH radical scavenging assay	Yes-HPLC
Wongsa et al. (2012)	Chiangrai province, Thailand	5	Yes- TPC, DPPH radical scavenging and antioxidant protection factor assays	Yes-HPLC
Thent and Das (2015)	Negeri Sembilan, Malaysia	5	Yes-Protocol citation	No
Luangpirom et al. (2014)	Khon Kaen Province, Thailand	20	Yes-Protocol citation	No
Hussan et al. (2013)	Selangor, Malaysia	10	Yes-Protocol citation	No
Thent et al. (2012a)	Negeri Sembilan, Malaysia	5	Yes-Protocol citation	No
Thent et al. (2012b)	Negeri Sembilan, Malaysia	5	Yes-Protocol citation	No
Peungvicha et al. (1998)	Bangkok, Thailand	30	No	No

DPPH, 2,2-diphenyl-1-picrylhydrazyl, FRAP, Ferric-reducing antioxidant power activity; HPLC, high performance liquid chromatography; UPLC, ultra performance liquid chromatography; TPC, total phenolic content.

### 3.4 Effect of *Piper sarmentosum* Roxb. on hypertension

There were six *in vivo* animal studies that focused on the effect of PS on hypertension (Mohd Zainudin et al., 2015; Alwi et al., 2018; Fauzy et al., 2019; Mohd Zainudin et al., 2019; Ugusman et al., 2020; Firdaus Azmi et al., 2021). Systolic blood pressure (SBP), diastolic blood pressure (DBP), mean arterial pressure (MAP) and nitric oxide (NO) levels were the most common parameters measured. All six studies showed that treatment with PS caused a marked reduction in the SBP, DBP and MAP of hypertensive rat models. A study by Fauzy et al. (2019) demonstrated that PS increased NO and reduced ET-1 levels in the mesenteric artery of SHR. Mohd Zainudin et al. (2015) showed that PS possessed an antihypertensive effect by reducing MDA and increasing serum NO levels in SHR. These findings were further supported by three other studies which demonstrated that the BP-lowering effect of PS was associated with increased NO levels in l-NAME-induced hypertensive rats (Alwi et al., 2018), dexamethasone-induced hypertensive rats (Ugusman et al., 2020; Firdaus Azmi et al., 2021) and SHR (Mohd Zainudin et al., 2019). Ugusman et al. (2020) found that PS increased vascular NO production by increasing eNOS mRNA expression, eNOS protein and eNOS activity in dexamethasone-induced hypertensive rats. Meanwhile, Mohd Zainudin et al. (2015) demonstrated that PS significantly decreased plasma ADMA levels in SHR. However, none of the studies investigated the effect of PS on target organ damage in hypertension.

### 3.5 Effect of *Piper sarmentosum* Roxb. on diabetes mellitus

Out of the 14 studies, eight studies assessed the effect of PS on diabetes mellitus. The effects of PS on diabetes were evaluated in two *in vitro* studies (Wongsa et al., 2012; Sallehuddin et al., 2020) and six *in vivo* studies (Peungvicha et al., 1998; Thent et al., 2012a; Thent et al., 2012b; Hussan et al., 2013; Luangpirom et al., 2014; Thent and Das, 2015). The *in vitro* studies involved screening of antidiabetic activity of PS using α-glucosidase and α-amylase inhibition assays. Wongsa et al. (2012) showed that PS had α-glucosidase but not α-amylase inhibitory activity. In contrast, another *in vitro* study revealed that PS had no α-glucosidase inhibitory activity (Sallehuddin et al., 2020). As for *in vivo* study, it was first reported that administration of 0.125 g/kg PS for 7 days significantly decreased blood glucose levels in both normal and diabetic rats (Peungvicha et al., 1998). Subsequently, Thent et al. (2012b) showed that PS increased the body weight and reduced fasting blood glucose and urine glucose levels in STZ-induced diabetic rats. Meanwhile, Luangpirom et al. (2014) found that diabetic rats treated with PS improved pancreatic islet function and increased serum insulin, leading to reduced fasting blood glucose.

#### 3.5.1 *Piper sarmentosum* Roxb.’s effect on diabetic complications/target organ and tissue damage

PS were also beneficial in attenuating the degenerative changes of the target organs in diabetes such as the heart, aorta, kidney, and liver. Light microscopic observation showed that PS reduced the degenerative changes in the myocardium and aortic tissues of diabetic rats as evidenced by lack of connective tissue deposit in the myocardium, reduced tunica media thickness, reduced tunica intima to tunica media ratio and less disruption of elastic fibre in the tunica media layer of the aorta (Thent et al., 2012a). Electron microscopic analysis further supported the findings as PS supplementation restored the ultrastructural integrity of the heart and aorta of diabetic rats (Thent et al., 2012b). As for the liver, PS increased the liver weight and reversed the diabetes-induced degenerative changes in the liver tissues as evidenced by the absence of nuclear deformation in the hepatocytes, less hyperemic areas in the sinusoids and less necrosis and vacuolization in the liver (Thent and Das, 2015). Interestingly, Hussan et al. (2013) demonstrated that PS prevented further progression of diabetic nephropathy in STZ-induced rats as evidenced by less contracted glomeruli, mild inflammatory cells infiltration, reduced urinary space size and absence of glomerular membrane thickening.

### 3.6 Meta-analysis

Only three studies were included in our meta-analysis, all of which provided data for the pooling of effects of PS on blood pressure parameters. No suitable data on glycemic parameters was available from the included studies, hence no meta-analysis was performed to explore the effects of PS on diabetes. The corresponding author (Luangpirom et al., 2014) was contacted to enquire for more details of the data, but no response was received.

#### 3.6.1 Effects of *Piper sarmentosum* Roxb. versus no treatment on hypertension

**FIGURE 5:**
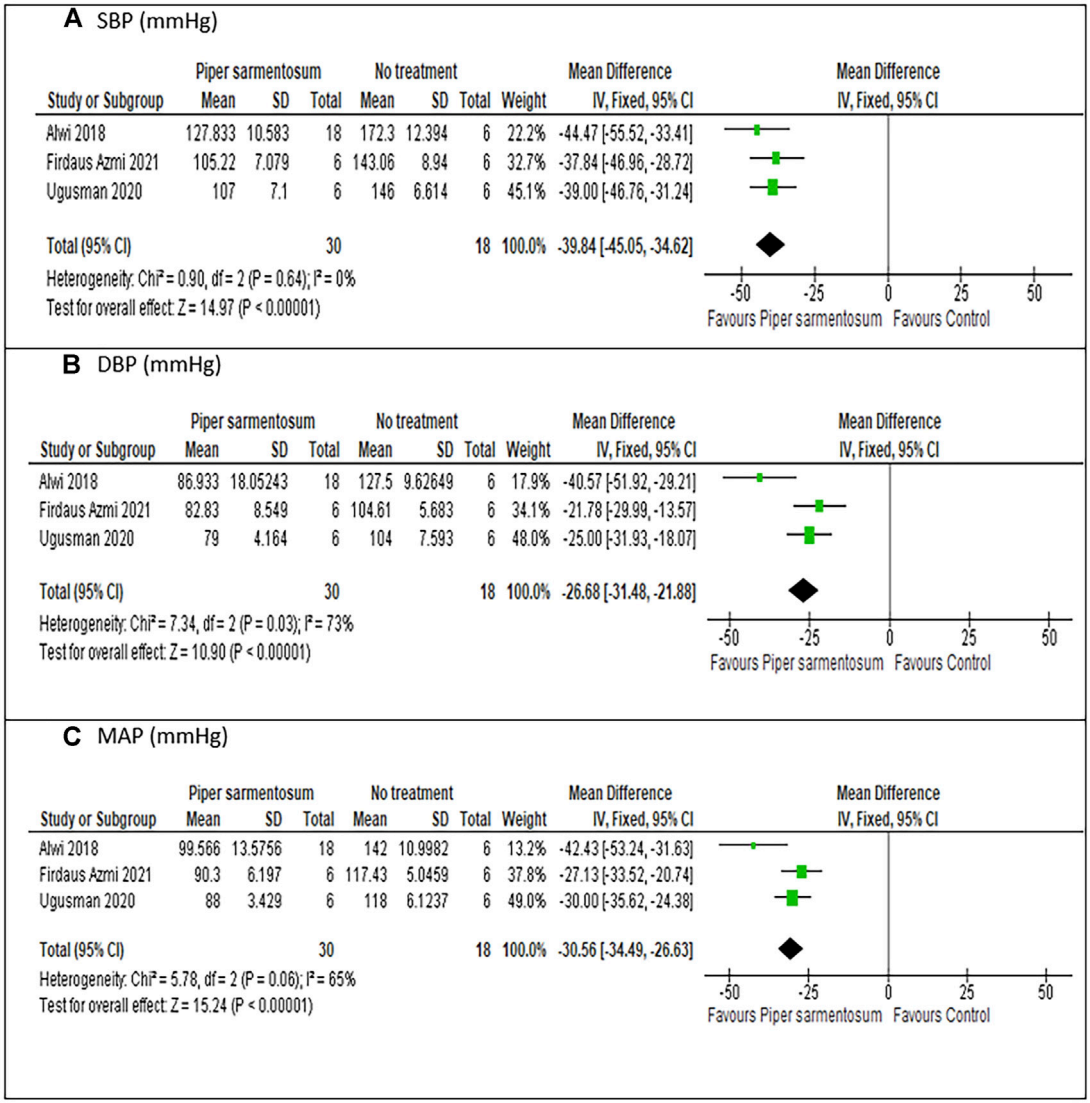
Meta-analysis of the effects of *Piper sarmentosum* Roxb. versus control on blood pressure: **(A)** SBP, Systolic blood pressure; **(B)** DBP, diastolic blood pressure, **(C)** MAP, mean arterial pressure.

**FIGURE 6:**
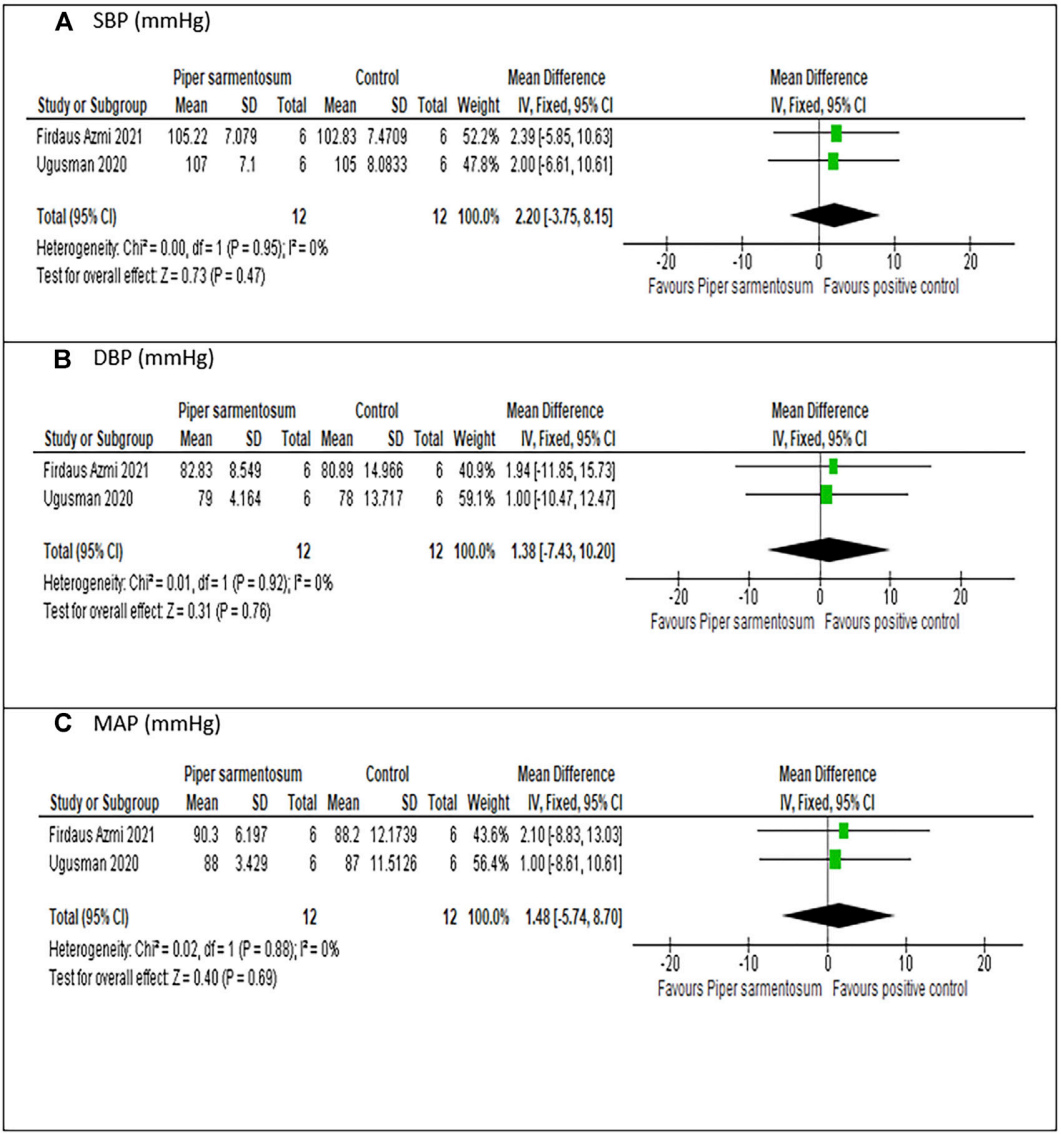
Meta-analysis of the effects of *Piper sarmentosum* Roxb. versus positive control on blood pressure: **(A)** SBP, Systolic blood pressure; **(B)** DBP, diastolic blood pressure, **(C)** MAP, mean arterial pressure.

Meta-analysis of three studies (Alwi et al., 2018; Ugusman et al., 2020; Firdaus Azmi et al., 2021) on the effect of PS versus no treatment have shown a statistically significant reduction in SBP with MD −39.84 mmHg (95% CI −45.05, −34.62; p < 0.01; Figure 5A) and no heterogeneity was observed. Similarly, pooling of results from three studies have shown that treatment with PS significantly reduced DBP and MAP with MD values of −26.68 mmHg (95% CI −31.48, −21.88; p < 0.01; Figure 5B), and -30.56 mmHg (95% CI −34.49, − 26.63; p < 0.01; Figure 5C) respectively, albeit the heterogeneity observed were substantial. A sensitivity analysis was performed to assess the robustness of PS effect on BP parameters. When limited to PS dose of 500 mg/kg, a comparable pooled effect size, direction, magnitude and statistical significance were obtained with MD −39.28 mmHg (95% CI −44.61, −33.95; p < 0.01; Figure 6A), −26.84 mmHg (95% CI −31.63, −22.05; p < 0.01; Figure 6B) and −29.74 mmHg (95% CI −33.82, −25.66; p < 0.01; Figure 6C) for SBP, DBP, and MAP respectively.

#### 3.6.2 Effects of *Piper sarmentosum* Roxb. versus positive control on hypertension

**FIGURE 7:**
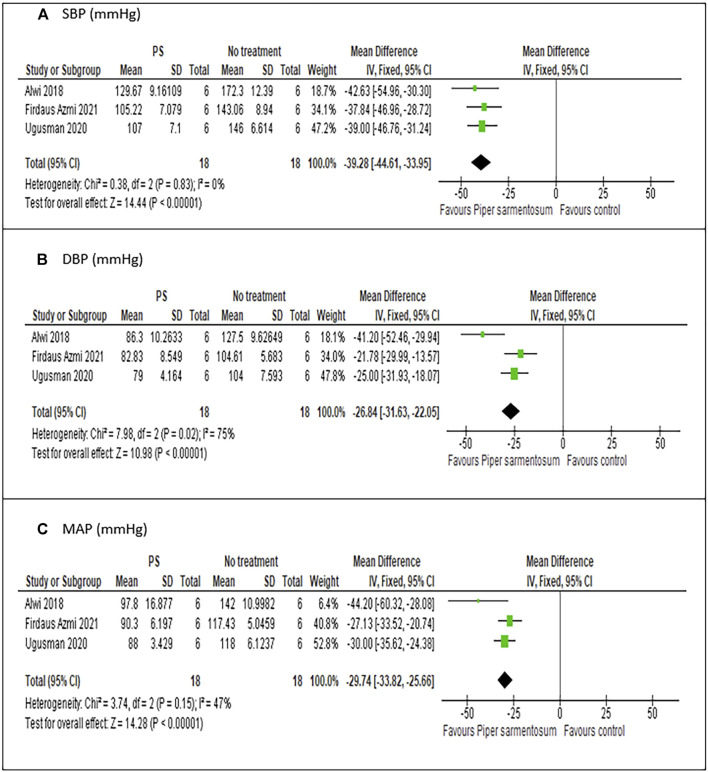
Sensitivity analysis of the effects of 500 mg/kg *Piper sarmentosum* Roxb. versus control on blood pressure: **(A)** SBP, Systolic blood pressure; **(B)** DBP, diastolic blood pressure, **(C)** MAP, mean arterial pressure.

Meta-analysis of three studies (Alwi et al., 2018; Ugusman et al., 2020; Firdaus Azmi et al., 2021) on the effect of PS versus captopril, a positive control, indicated no significant difference in SBP (MD = 2.20 mmHg, 95% CI −3.75, 8.15; p = 0.47; Figure 7A), DBP (MD = 1.38 mmHg, 95% CI −7.43, 10.20; p = 0.76; Figure 7B) and MAP (MD = 1.48 mmHg, 95% CI −5.74, 8.70; p = 0.69; Figure 7C). No heterogeneity was observed.

## 4 Discussion

The present systematic review and meta-analysis suggests that PS has beneficial effects on BP and glycemic control in hypertensive and diabetic animal models.

### 4.1 Effects of *Piper sarmentosum* Roxb. on hypertension

PS has demonstrated its antihypertensive effect in various hypertensive animal models including SHR (Mohd Zainudin et al., 2015; Fauzy et al., 2019; Mohd Zainudin et al., 2019), dexamethasone-induced hypertensive rats (Ugusman et al., 2020; Firdaus Azmi et al., 2021) and l-NAME-induced hypertensive rats (Alwi et al., 2018). Our meta-analysis has shown that PS supplementation resulted in significantly reduced SBP, DBP, and MAP by 39.84, 26.688, and 30.56 mmHg, respectively, in hypertensive rat models. The significant reduction is only observed when the groups of hypertensive animals receiving PS were compared to hypertensive rats that do not receive any intervention. No significant difference was observed in BP parameters when the groups of hypertensive animals receiving PS were compared to the groups of animals receiving positive control. A positive control is used in the included studies to control for variability of the experiments as well as for unbiased and objective observation of the effects of studied intervention on BP (Torday and Baluška, 2019). Although the mechanism involved may be different, the analysis has shown evidence that PS has similar effects, if not equal, to captopril in reducing BP parameters.

SHR exhibits many features of human essential hypertension, including increased blood pressure and total peripheral resistance. These features are preceded by oxidative stress, which leads to endothelial dysfunction (Fauzy et al., 2019). MDA, a lipid peroxidation product, is a marker of oxidative stress damage. PS administration to SHR caused a significant reduction in MDA level (Mohd Zainudin et al., 2015), suggesting that the antihypertensive effect of PS was attributed to its antioxidative activity. A previous study also demonstrated that PS reduced oxidative stress by decreasing MDA in endothelial cells (Hafizah et al., 2010). Interestingly, another study showed that PS enhanced the expression of the antioxidant enzymes; superoxide dismutase (SOD), catalase (CAT) and glutathione peroxidase in hydrogen peroxide-induced endothelial cells (Ugusman et al., 2011).

A healthy endothelium releases a plethora of vasoactive factors that contribute to maintaining vascular homeostasis. Endothelial vasoactive mediators such as NO and ET-1 can either decrease or increase the vascular tone, respectively. The imbalance between endothelium-derived vasodilators and vasoconstrictors leads to endothelial dysfunction, which is a precursor of hypertension (Rubanyi, 1993). Endothelial dysfunction occurs when there is reduced NO synthesis, decreased NO bioavailability, or NO antagonism by endothelium-derived vasoconstrictors such as ET-1 (Mordi et al., 2016). The majority of vascular NO is synthesized by eNOS. Meanwhile, decreased bioavailability of NO can be attributed to reactive oxygen species (ROS) that converts NO to peroxynitrite (Mordi et al., 2016).

Dexamethasone-induced hypertensive rats showed reduced eNOS mRNA expression, protein, activity, and serum NO level (Ugusman et al., 2020). Several studies have reported that dexamethasone results in overproduction of ROS that decreases NO bioavailability, which in turn leads to hypertension (de Gennaro Colonna et al., 2002; Rojas et al., 2006; Dubey et al., 2017). Dexamethasone also increases the level of angiotensin-converting enzyme, which is responsible for increasing blood pressure (Fishel et al., 1995). Meanwhile, chronic blockade of NO synthesis by l-NAME is a well-known model of experimental hypertension. It is well established that administration of l-NAME inhibits NO synthesis, causes endothelial dysfunction and vasoconstriction, and thus leads to hypertension (Alwi et al., 2018).

Supplementation of PS successfully reduced the blood pressure of dexamethasone-induced and l-NAME-induced hypertensive rats. This could be attributed to the positive effect of PS on NO production (Alwi et al., 2018; Ugusman et al., 2020; Firdaus Azmi et al., 2021). PS treatment in dexamethasone-induced hypertensive rats increased the eNOS mRNA level, protein level and activity and NO level (Ugusman et al., 2020). Upregulation of eNOS mRNA expression causes more eNOS protein to be synthesized, leading to increased eNOS activity and NO generation. These findings were congruent with a previous study whereby PS increased NO production in hydrogen peroxide-induced endothelial cells by increasing eNOS mRNA expression, protein level and activity (Ugusman et al., 2010). Owing to the antioxidative activity of PS, PS may increase the bioavailability of NO (Mohd Zainudin et al., 2019), as an antioxidant protects NO from the breakdown by ROS (d’Unienville et al., 2021). In SHR, oral administration of a potent antioxidant, lazaroid, improves NO bioavailability and reduces blood pressure (Sorriento et al., 2018).

Competitive inhibitor of eNOS such as ADMA lowers NO synthesis (Hsu and Tain, 2021). Decreased NO also gives rise to unabated ET-1 actions that contribute to vasoconstriction, thereby increasing blood pressure (Böger and Ron, 2005). SHR showed increased ADMA level, which was attenuated by PS supplementation (Mohd Zainudin et al., 2019). Decreased ADMA level is associated with its effective degradation by dimethylarginine dimethylaminohydrolase (DDAH). A previous study reported that PS stimulated DDAH activity in tumor necrosis factor-α-induced endothelial cells, which in turn reduced ADMA level and enhanced NO production (Sundar et al., 2019). Apart from stimulating NO production, PS also reduced ET-1 level in the mesenteric artery of SHR (Fauzy et al., 2019). This observation indicates that PS possesses an anti-vasoconstrictor effect, which contributes to its antihypertensive action.

Even though all the studies reviewed showed positive effects of PS on hypertension, all the studies are animal studies with no clinical trials performed on hypertensive patients. Besides, none of the studies investigated the effect of PS on the renin-angiotensin system, which is an important system that regulates blood pressure. Only two studies on the effect of PS on hypertension that identified the active compounds present in their PS extracts, in which the extracts contain rutin and vitexin (Ugusman et al., 2020; Firdaus Azmi et al., 2021). Oral administration of rutin lowers the blood pressure of l-NAME-induced hypertensive rats (Ganga Raju et al., 2019). Other studies involving phytochemical analysis of PS showed that PS also contains piperine, myricetin and quercetin (Firdaus Azmi et al., 2021). Piperine reduces the blood pressure of l-NAME-induced hypertensive rats (Kumar et al., 2010). Myricetin has also been reported to lower the blood pressure of deoxycorticosterone acetate-salt-hypertensive rats (Borde et al., 2011), whereas quercetin lowers the blood pressure of SHR (Galindo et al., 2012).

### 4.2 Effects of *Piper sarmentosum* Roxb. on diabetes mellitus

Hyperglycemia is the hallmark of diabetes secondary to either insufficient insulin secretion, resistance to the action of insulin, or both. Induction of diabetes in laboratory animals using chemical ablation of pancreatic β-cells is the most common experimental model. Alloxan and STZ are the most popular diabetogenic chemicals in diabetes research. However, STZ has notable advantages over alloxan due to its rapid onset, the long half-life, low toxicity and cost-effectiveness (Wszola et al., 2021). STZ is a toxic glucose analogue that is preferentially accumulated in the pancreatic β-cells via the GLUT2 glucose transporters in the plasma membrane.

Following its uptake into beta-cells, STZ triggers oxidative stress, eventually causing the pancreatic β-cells to undergo necrotic cell death, resulting in hypoinsulinemia and hyperglycemia (Choudhari et al., 2017). Additionally, GLUT2 transporters are not only expressed in pancreatic β-cells, but also in the epithelial cells of the kidneys and hepatocytes (Sędzikowska and Szablewski, 2021; Yang et al., 2022). Thus, administration of STZ may result in nephro- and hepatotoxicity along with its potential to damage pancreatic β-cells (Wszola et al., 2021). All six animal studies that investigated the effects of PS on diabetes used a single, high dose of STZ injection (up to 75 mg/kg) to induce pancreatic β-cells damage and diabetes in rats, which mimics type 1 diabetes. None of the studies incorporates the rodent models of type 2 diabetes with underlying insulin resistance, obesity and pancreatic dysfunction, such as the Zucker diabetic fatty rat and db/db mouse (Schwarzer, 2016).

PS supplementation reduced the fasting blood glucose levels (Peungvicha et al., 1998) and urine glucose levels (Thent et al., 2012b) of STZ-induced diabetic rats. One of the mechanisms of the glucose-lowering effect of PS is through improved pancreatic islet function and increased serum insulin (Luangpirom et al., 2014). However, in one study, PS did not cause any significant reduction in the diabetic rats’ fasting blood glucose levels (Hussan et al., 2013). The authors only used fasting blood glucose to measure the rats’ glycemic status without other supporting tests such as the oral glucose tolerance test and HbA1c, thus making the glycemic status assessment less accurate (Bur et al., 2003). Besides, a reduction in body weight was observed in rats following diabetes induction with STZ (Thent et al., 2012b; Hussan et al., 2013). In response to hypoinsulinemia, the body starts burning fat and muscle for energy, causing a reduction in overall body weight (Eleazu et al., 2013). However, the body weights of the diabetic rats were restored with PS supplementation (Thent et al., 2012b). Since PS has been shown to improve pancreatic islet function and increase serum insulin (Thent et al., 2012b; Luangpirom et al., 2014), this might contribute to the improved body weight of the diabetic rats.

PS also exhibits an α-glucosidase inhibitory effect, but not an α-amylase inhibitory effect (Wongsa et al., 2012). In contrast, another *in vitro* study showed that PS did not possess any α-glucosidase inhibitory effect (Sallehuddin et al., 2020). Alpha-glucosidase and α-amylase inhibitory effects are commonly used to screen the antidiabetic action of natural products (Kittiwisut et al., 2021). Alpha-glucosidase inhibitors act by inhibiting the enzyme α-glucosidase, such as glucoamylase, sucrase, maltase, and isomaltase at the brush border of the intestinal epithelium. This action will block the absorption of carbohydrates in the small intestine, hence reducing postprandial hyperglycemia (Malunga et al., 2016). Meanwhile, α-amylase inhibitors prevent the hydrolysis of α-(1–4)-d-glucosidic linkages in starch, thus reducing carbohydrate digestion and absorption in the gastrointestinal tract and lowering blood glucose (Gong et al., 2020). Most plant-based natural products were effective against either α-amylase or α-glucosidase, with just a few exceptions being effective against both enzymes (Poovitha and Parani, 2016). Besides, α-amylase or α-glucosidase inhibitory assays serve as screening tools for the extract’s antidiabetic activity, in which the results should be further supported by *in vivo* study findings.

Among the target organs of diabetic complications are the kidney, liver, heart and blood vessels (Wei et al., 2022). PS has been proven to attenuate the damaging effects of diabetes in the kidney, liver, heart and aorta (Thent et al., 2012a; Thent et al., 2012b; Hussan et al., 2013; Thent and Das 2015). Significant alterations in the liver weight, histology and oxidative stress markers are linked to STZ-induced diabetes (Rodríguez et al., 2018; Yazdi et al., 2019). Treatment with PS also increased the liver weight and reversed diabetes-induced degenerative changes in the liver tissues; an effect attributed to its antioxidative action (Thent and Das, 2015). Inflammation in response to intermittent or chronic hyperglycemia is involved in the initiation and progression of diabetic nephropathy (Amorim et al., 2019; Donate-Correa et al., 2021). Diabetic rats have increased kidney weight due to renal hypertrophy, with histological changes of diabetic nephropathy such as contracted glomeruli with widened urinary spaces, marked inflammatory cell infiltration in the renal cortex and medulla, and glomerular membrane thickening (Hussan et al., 2013). Even though treatment with PS did not change the kidney weight, it attenuated the histological changes in the diabetic rat’s kidney as evidenced by less contracted glomeruli, mild inflammatory cells infiltration, reduced urinary space size and absence of glomerular membrane thickening. This could be due to the anti-inflammatory effect of PS (Hussan et al., 2013). Furthermore, PS supplementation reduced the degenerative changes and restored the ultrastructural integrity of the heart and aorta of diabetic rats under light and electron microscopic analyses (Thent et al., 2012a; Thent et al., 2012b). Overall, PS attenuates the complications of diabetes in the kidney, liver, heart and blood vessels through its antioxidative, anti-inflammatory and glucose-lowering effects.

Even though most of the studies reviewed showed positive effects of PS on diabetes, all studies are preclinical studies with no clinical trials performed on diabetic patients. Besides, none of the studies used type 2 diabetes model, which is more common in the society. Only two studies on the effect of PS on diabetes that identified the active compounds present in their PS extracts, in which the extracts contain catechin, naringin (Sallehuddin et al., 2020), caffeic acid and p-coumaric acid (Wongsa et al., 2012). Treatment with catechin lowers blood glucose levels and increases the activity of antioxidant enzymes such as SOD, CAT and glutathione-S-transferase in diabetic rats (Gong et al., 2020). In another study, naringin administration decreased the plasma glucose of diabetic rats as measured using OGTT (Ahmed et al., 2012). Caffeic acid reduces fasting blood glucose when given to alloxan-induced diabetic rats (Oršolić et al., 2021), whereas p-coumaric acid improves glycemic status and increases plasma insulin level in STZ-induced diabetic rats (Amalan and Vijayakumar, 2015).

### 4.3 Strength and limitation of the study

To the best of our knowledge, this is the first systematic review and meta-analysis investigating the effects of PS on hypertension and diabetes. The systematic literature search ensures all relevant articles were identified whilst at the same time minimizes selection bias. In addition, the meta-analyses performed enables objective evaluation of the effect of PS on BP parameters. However, the current review is not without its limitation. The small number of included studies may have influenced the effect estimates generated in our meta-analysis. Therefore, interpretation of results should be made with caution. Moreover, the various approach used in reporting the results did not allow us to objectively report the effect of PS on glycemic control. Nevertheless, most of the studies reported uniform effects of PS on diabetes mellitus.

## 5 Conclusion

Overall, PS showed promising antihypertensive and antidiabetic effects. However, all the studies reviewed are preclinical studies with no randomized clinical trials conducted to investigate the antihypertensive and antidiabetic effects of PS in human subjects. Therefore, further clinical studies are recommended to validate the antihypertensive and antidiabetic activities of PS to prepare evidence-based formulations.

## Data Availability

The original contributions presented in the study are included in the article/Supplementary Material, further inquiries can be directed to the corresponding authors.
